# Patient advocacy and DSM-5

**DOI:** 10.1186/1741-7015-11-133

**Published:** 2013-05-17

**Authors:** Dan J Stein, Katharine A Phillips

**Affiliations:** 1 Department of Psychiatry, University of Cape Town and Groote Schuur Hospital J2, Anzio Rd, Observatory 7925, Cape Town, South Africa; 2 Rhode Island Hospital and the Department of Psychiatry and Human Behavior, Alpert Medical School of Brown University, Coro Center West, Suite 2.030, 1 Hoppin Street, Providence, RI, 02903, USA

**Keywords:** Diagnostic and Statistical Manual of Mental Disorders, Psychiatric classification, Nosology, Diagnosis, Patient advocacy, Consumer advocacy, Obsessive-compulsive and related disorders

## Abstract

The revision of the Diagnostic and Statistical Manual of Mental Disorders (DSM) provides a useful opportunity to revisit debates about the nature of psychiatric classification. An important debate concerns the involvement of mental health consumers in revisions of the classification. One perspective argues that psychiatric classification is a scientific process undertaken by scientific experts and that including consumers in the revision process is merely pandering to political correctness. A contrasting perspective is that psychiatric classification is a process driven by a range of different values and that the involvement of patients and patient advocates would enhance this process. Here we draw on our experiences with input from the public during the deliberations of the Obsessive Compulsive-Spectrum Disorders subworkgroup of DSM-5, to help make the argument that psychiatric classification does require reasoned debate on a range of different facts and values, and that it is appropriate for scientist experts to review their nosological recommendations in the light of rigorous consideration of patient experience and feedback.

## Introduction

The current revision of the Diagnostic and Statistical Manual of Mental Disorders (DSM) provides a useful opportunity to revisit a broad range of debates about the nature of psychiatric classification, and indeed of psychiatry. Some of the debate has understandably focused on the extent to which advances in basic and clinical neuroscience can and should inform revisions of the classification [1,2]. There has perhaps been less debate on the extent to which advances in public health constructs and knowledge can and should inform such revisions [3].

In this paper, we want to consider a somewhat orthogonal set of considerations – namely, the question of who should be included in deliberations about revisions of the classification. There has been some debate about the inclusion of different professional groups and individuals from different locations, and it is gratifying to see the substantial extent to which DSM-5 has been a multidisciplinary and multinational process [4]. There has perhaps been less debate about the extent to which lay perspectives should also be included, although the DSM-5 leadership has emphasized their importance [5].

We begin by outlining three contrasting approaches to the debate about the inclusion of patient, consumer, client, or health care user perspectives during the revision of psychiatric diagnostic classifications. We then consider the responses of the public to recommendations put forth by the DSM-5 subworkgroup on Obsessive Compulsive-Spectrum Disorders (which oversaw revisions for the chapter of Obsessive-Compulsive and Related Disorders in DSM-5). We conclude by arguing that it is important for scientific experts to review their nosological recommendations in the light of rigorous consideration of consumer experience and feedback.

### Approaches to classification

For heuristic purposes, it is useful to outline a ‘classical’ approach to psychiatric classification. This approach is based on particular philosophical positions on the nature of science, the mind and language (for example, positivism), and the idea that diagnostic entities can be defined by necessary and sufficient criteria [6,7]. The classical approach sees diagnostic entities as similar to squares, where the operational definition is invariant from time to time, and place to place. This approach is consistent with the view that science is a process of obtaining data from observations of the world and of developing laws that describe the relationships between such data.

According to this view, the most relevant people to consult when revising a diagnostic category are those who are scientific experts in the relevant area. Scientific experts are familiar with, or have generated, the relevant data which speak to the optimal diagnostic criteria. Over time, the diagnostic criteria more closely approximate the true nature of the relevant entity. Increasingly, the hope is stated that the diagnostic criteria will include particular biomarkers [1].

For heuristic purposes, it is useful to also outline a ‘critical’ approach to psychiatric classification. This approach is based on philosophical positions on the nature of science, the mind and language which contrast with the classical ones (for example, hermeneuticism), and the idea that psychiatric entities reflect the values of classifiers [6,7]. The critical approach sees diagnostic entities as similar to weeds, which are defined differently by different classifiers from time to time and from place to place. This approach is consistent with the view that science, and particularly social science, is a social process which involves participation in the world and an understanding of human activities.

According to this view, the question of whom to include when revising a psychiatric classification is crucially important. Different classifiers may bring to bear different sets of expertise and different kinds of values. It may, therefore, be relevant to include in the process not only a range of professionals, but also those to whom the classification will be applied. Terms such as patients, clients, health care users, and consumers each convey their own set of values, but for simplicity’s sake we use ‘patients’ here.

An integrative perspective is a third approach, which relies on philosophical constructs that draw on both the classical and critical positions. This approach holds that categories reflect both human practices as well as real underlying structures and mechanisms. Weeds, for example, are plants that are not wanted by gardeners, and they also have typical characteristics (such as smothering other plants). While psychiatric classification requires the weighing of a range of facts and values, this can be done in a reasonable way, so that advances in the nosology are possible [6,7].

According to the integrative view, it is important to consider a range of relevant facts and values when revising the psychiatric nosology. Diagnostic entities should have both clinical utility and diagnostic validity, and over time we can develop categories with greater utility and validity. While considerations about clinical utility and diagnostic validity are ultimately weighed by scientific experts, this weighing should include a rigorous knowledge and understanding of patients’ experience. We consider the integrative perspective the most valuable, and we next draw on experiences during the development of DSM-5 that reflect this view.

### Getting input on obsessive-compulsive and related disorders

The DSM-5 development process differed substantially from the development of prior editions of DSM in a number of ways. One of the most notable differences was the extensive feedback that was solicited from the public regarding proposed changes to DSM-IV. This effort was facilitated by the availability of the internet, which was not yet widely available when DSM-IV was developed during the early 1990s. Changes proposed for DSM-5 by the various workgroups were posted three times on the DSM-5 website in order to solicit input from a broad range of individuals. The first posting alone received nearly 40 million hits and more than 8,000 comments from professionals, consumers, and interested individuals [5].

Our DSM-5 subworkgroup on Obsessive-Compulsive Spectrum Disorders pondered a range of questions about the optimal classification of these conditions. A first question was whether DSM-5 should include a separate section on obsessive-compulsive and related disorders. A second question was whether new entities such as hoarding disorder and excoriation (skin picking) disorder, which were not included in prior editions of DSM, should be included in this section. A third question concerned whether some entities, such as trichotillomania, should have a different name. A fourth question concerned the optimal diagnostic criteria for entities in this section.

Our subworkgroup reviewed the literature on obsessive-compulsive and related disorders [8], and concluded that there was sufficient scientific evidence to support the utility and validity of this construct, which has been widely adopted by the field for more than a decade. At the same time, our review of the literature on anxiety disorders and obsessive-compulsive disorder suggested that there was considerable overlap between these sets of conditions [9]. The final recommendation of our subworkgroup was that anxiety disorders and the new chapter of obsessive-compulsive and related disorders should be placed next to each other in the nomenclature in order to emphasize the close relationship between some of the disorders in these two chapters - for example, between body dysmorphic disorder and social anxiety disorder (social phobia) [10].

There was considerable feedback from the public, via the internet postings, on this question. Patient advocates for Tourette’s Disorder were concerned about the possibility that Tourette’s Disorder would be classified in the anxiety disorders’ chapter or the obsessive-compulsive and related disorders’ chapter. They argued that it was important that Tourettte’s Disorder be seen as a neurodevelopmental condition, and that it, therefore, fit best into the new chapter of neurodevelopmental disorders. Patient advocates for trichotillomania and skin picking disorder were concerned that if these disorders were classified alongside obsessive-compulsive disorder, patients might receive inappropriate treatment.

In considering these viewpoints, our subworkgroup noted that there were both overlaps and distinctions between OCD and a range of other putative obsessive-compulsive and related disorders, and that this was the case in other DSM-5 chapters as well. We also noted that a tic specifier for obsessive-compulsive disorder was important, as OCD patients with tics have particular clinical characteristics, and that adding a tic specifier to obsessive-compulsive disorder would emphasize the clinically important relationship between tic disorders and OCD [11]. Finally, our subworkgroup noted that it would be important to use the DSM-5 text to emphasize to readers how disorders in the chapters on obsessive-compulsive and related disorders had both similarities and differences.

Our subworkgroup reviewed the literature on hoarding disorder and excoriation (skin picking) disorder [12,13] and concluded that there was sufficient scientific evidence to support the utility and validity of these diagnostic entities. (We should note that all changes proposed by the subworkgroup received extensive review by other DSM-5 committees to ensure that the proposed changes had adequate scientific support, as changes for DSM-5 were heavily based upon available scientific evidence.) There was again considerable feedback from the public, including members of the Trichotillomania Learning Center, on these questions. In particular, hundreds of patients and patient advocates commented that excoriation (skin picking) disorder and hoarding disorder should be included in the nomenclature. Such feedback usefully complemented available scientific data in emphasizing the clinical importance and the clinical utility of these diagnostic entities.

Our subworkgroup carefully considered whether the term ‘trichotillomania’, which was used in DSM-IV, was an optimal name for this condition. We noted the concerns of some patient advocates that ‘mania’ could be perceived as pejorative and inaccurate. At the same time, we noted the concerns of other patient advocates that the alternative term ‘hair pulling disorder’, because of its somewhat colloquial nature, could possibly detract from the disorder’s medical significance. Our subworkgroup also took into account the view that a change in name could lead to confusion in the scientific literature. Our subworkgroup, therefore, recommended a compromise name: ‘trichotillomania (hair pulling disorder)’. A similar set of considerations led to the choice of the term ‘excoriation (skin picking) disorder’. Our subworkgroup considered the scientific literature on diagnostic criteria for the various entities in the chapter on obsessive-compulsive and related disorders. We noted, for example, that the criteria for trichotillomania were based on the notion that this was an impulse control disorder, and that a sizeable minority of patients with distressing/impairing and chronic hair-pulling did not demonstrate all of the DSM-IV criteria. Comments on the website strongly agreed with our subworkgroup’s recommendation to change the problematic criteria; this change was also supported by a study sponsored by the subworkgroup that tested alternative criteria [14].

In summary, feedback from patients and patient advocates played a useful role in influencing some of our subworkgroup recommendations for the DSM-5 section on obsessive-compulsive and related disorders. This feedback complemented and, in some cases, reinforced the value of recommendations that were based on the scientific literature. Perhaps the most helpful impact of this feedback was support from many individuals who suffered from excoriation (skin picking) disorder to add this condition to DSM-5; this input usefully supported the view that the available scientific data were sufficient to add this disorder to DSM-5. This feedback was also helpful in assisting our subworkgroup with making recommendations for which no scientific data were available on which to base a recommendation (such as the best name for trichotillomania).

## Conclusion

Our view, consistent with the integrative position, is that psychiatric classification requires reasoned debate on a range of different facts and values, and that it is, therefore, appropriate for scientific experts to review their evidence-based nosological recommendations in the light of rigorous consideration of patient experience and feedback. Given the complexity of defining psychiatric disorders and their boundaries [15,16], any particular approach to the nosology has both pros and cons [17], and reaching an optimal solution is enhanced by carefully considering a broad range of perspectives [18], including the views of patients and patient advocates [19].

It is notable that previous authorities have differed considerably about the importance of including patients in the process of revising psychiatric classifications [20,21]. Such disagreement perhaps reflects differences on which values to emphasize during the process of classification revision [22,23]. Nevertheless, it does appear that there is growing consensus among nosologists, clinicians, and researchers that psychiatric diagnosis and treatment is necessarily dependent on first-person accounts of subjective experience [24-26], that understanding and appreciating individual voices and variations is crucial [27], and that it is important to include people with or in recovery from mental illness as collaborators in clinical and research processes [28].

That said, there is a dearth of data on questions such as whether patient involvement in developing healthcare policy and research, clinical practice guidelines and patient information material is, in fact, helpful, and rigorous study of such involvement is needed [29]. Similarly, while different models of patient involvement in nosology have been proposed and developed [30], much additional work is needed to compare carefully such models and their impacts. Furthermore, it needs to be kept in mind that even if a diagnostic system incorporates patient perspectives, assessment and intervention should proceed with appropriate care in order to diminish stigma [31,32].

Our hope is that the addition of a chapter on obsessive-compulsive and related disorders, including hoarding disorder and excoriation (skin picking) disorder, has more pros than cons and will be useful for individuals living with these conditions, and for their care. The DSM-5 development process had very rigorous guidelines and criteria for adding new disorders to DSM-5; enough scientific evidence was available on both conditions to support their addition to DSM-5. However, the input of patients and patient advocates usefully highlighted and supported the clinical importance of adding these disorders to the nomenclature.

Our hope also is that the diagnostic criteria for these entities, and all of those in the chapter of obsessive-compulsive and related disorders, are as optimal as possible. Many patients and patient advocates were supportive of the changes that we proposed and that were ultimately accepted during the extensive review process of workgroup proposals. This is reassuring, as patients have an intimate knowledge of the experience of suffering from particular disorders, and so may well be able to shed useful light on particular diagnostic criteria. Further editions of DSM should continue to encourage feedback from the public, and scientific experts should continue to pay close attention to this valuable input as they develop their recommendations.

## Competing interests

DJS has received research grants and/or consultancy honoraria from Abbott, Astrazeneca, Biocodex, Eli-Lilly, GlaxoSmithKline, Jazz Pharmaceuticals, Johnson & Johnson, Lundbeck, Orion, Pfizer, Pharmacia, Roche, Servier, Solvay, Sumitomo, Takeda, Tikvah, and Wyeth. Katharine A. Phillips Financial Disclosure includes receipt of salary and/or research support in the past 12 months from the National Institute of Mental Health (salary and research funding), Forest Laboratories (medication only for a study sponsored and funded by the National Institute of Mental Health) and Transcept Pharmaceuticals (research funding). Katherine A Phillips provided Consulting for Janssen Research and Development and received honoraria, royalties, or travel reimbursement from Oxford University Press, Guilford Press, Merck Manual (future) and Up to Date (future). She also received speaking or grant reviewing honoraria and/or travel reimbursement from academic and federal institutions and from professional organizations and the free pass from potential future royalties.

## Authors’ information

DJS served as Chair of the DSM-5 subworkgroup on obsessive-compulsive spectrum disorders. KAP served as Chair of the DSM-5 workgroup on anxiety, obsessive-compulsive spectrum, posttraumatic and dissociative disorders.

## Pre-publication history

The pre-publication history for this paper can be accessed here:

http://www.biomedcentral.com/1741-7015/11/133/prepub
